# Will the zero-margin drug policy reduce the economic burden of stroke patients in China?

**DOI:** 10.7189/jogh.11.08007

**Published:** 2021-09-30

**Authors:** Quan Fang, Degao Shang, Yunxia Zhang, Xinli Geng, Fang Liu, Qin Zhang, Xin Wang

**Affiliations:** 1 College of Health Management, China Medical University, Shenyang, Liaoning, China; 2 Liaoning Province Center for Disease Control and Prevention, Shenyang, Liaoning, China; 3 Institute for Shanxi Medical Institution Administration, Taiyuan, Shanxi, China; 4 Shengjing Hospital of China Medical University, Shenyang, Liaoning, China; 5 Research Center for Health Development – Liaoning New Type Think Tank for University, Shenyang, Liaoning, China

## Abstract

**Background:**

China is facing a more severe stroke challenge. In 2017, China fully implemented the zero-margin drug policy (ZMDP), cancelling 15% of drug markups in public hospitals. Based on the “System of Health Accounts 2011” (SHA 2011), this paper explores the changes in the economic burden of stroke in China after implementing ZMDP to provide an accurate reference for policymakers.

**Methods:**

Stroke patients from 2016 to 2018 were selected by multistage stratification probability-proportional random sampling in Shanxi Province. A total of 223 187 samples were included. Regression discontinuity design (RDD) was used to measure the change of drug proportion and cost. Sensitivity analysis and subgroup analysis were implied to determine the stability of the results.

**Results:**

The current curative expenditure on stroke from 2016 to 2018 was 4374.69, 3727.22, and 3752.52 million Chinese Yuan (CNY). About 90% of the cost occurred during hospitalization, and 60% in general hospitals. After the implementation of ZMDP, the drug proportion from 46.54% (interquartile range (IQR) = 37.10%, 55.14%) in 2016 to 36.40% (IQR = 25.82%, 48.58%) in 2018, and average hospitalization cost per stay was from 7950.02 (IQR = 4938.76, 12 639.90) CNY in 2016 to 7362.08 (IQR = 4892.82, 11 501.40) CNY in 2018. RDD showed the drug proportion decreased by 2.76% (95% confidence interval (CI) = 1.17%, 4.35%, *P* = 0.001), and the expense decreased by 4698.34 (95% CI = 3047.59, 6349.09, *P* < 0.001) CNY.

**Conclusions:**

The economic burden of stroke patients in China was severe. ZMDP played a noticeable effect in reducing drug proportion and hospitalization costs. The Chinese government should continue to implement relevant policies to control stroke costs according to regional and population characteristics.

Stroke is a major public health problem worldwide. It is the second leading cause of death and the third leading cause of disability globally [1]. According to the Global Burden of Disease study, stroke has an approximately 25% risk starting at 25 years and causes 5.5 million deaths a year [2,3].

Stroke is a catastrophic illness often accompanied by long-term disability and other psychological problems [4]. For example, the incidence of depression is 22% [5], and dysphagia is between 37% to 78% among stroke survivors [6]. Besides, it can also be accompanied by various complications, such as hypertension, diabetes [7], which require long-term medication. Stroke accounts for an average of around 2%-4% of total health expenditure worldwide, and the ratio is higher in high-income countries [8]. Between 2015 and 2035, total direct medical stroke-related costs are projected to more than double in the USA, from US$ 36.7 billion to US$94.3 billion [9].

As the world's largest developing country, China is facing a more severe stroke challenge. In China, the onset age of stroke patients tends to be younger and younger, with a high proportion of patients under 50 years old. [10]. China already accounts for one-third of the world's stroke deaths, and stroke has risen from third place in 1990 to first place in 2017 on the impact of disability-adjusted life years [11]. At present, the average annual medical expense of stroke patients in China urban areas is 10 637 Chinese Yuan (CNY, 1 CNY ~ 0.1511 US$ in 2018), of which the drug cost accounts for more than 50% [12].

The key to reduce the economic burden of stroke is making the best decisions [13]. In recent years, China has taken a series of measures to control the health expenditure of stroke, such as improving insurance coverage and quality of care, which is a long cost-effective way to reduce the burden of stroke. Although some progress has been made, existing evidence suggests they have not entirely reversed the increased economic burden of stroke. As the primary source of economic burden for stroke patients, drug costs remain high [14]. The solution seems to be the zero-margin drug policy (ZMDP). It is an essential part of the new round of health care reform launched by the Chinese government in 2009 [15]. Before 2017, most hospitals in China could sell retail drugs at a price plus a 15% profit margin as a source of financing for health care providers [16]. To correct this distorted incentive to reduce the growing economic burden of disease, China began to promote the ZMDP. It requires the elimination of 15% profit on drug sales, thus changing the profit-making behavior of health care providers [17]. The ZMDP can reduce the drug proportion, but studies on the economic burden of this policy on stroke patients are lacking.

From a public health perspective, understanding the financing and distribution of stroke treatment costs is particularly important as they form the basis for understanding the economic challenges [18]. To reflect the economic burden of stroke patients in China, “System of Health Accounts 2011 Edition” (SHA 2011) was used in this study. SHA 2011 can reflect the flow of health costs from the three dimensions of financing, consumption, and production, increasing international and domestic comparability and meeting health system analysis and policymakers [19].

Previous studies about the economic burden of stroke are mainly concentrated in Europe, the United States, and other developed countries. The reference value for China is limited [20-22].

## METHODS

In this paper, the data of stroke patients in Shanxi Province, China, from 2016 to 2018 were selected to analyze the economic burden of stroke patients. At the same time, regression discontinuity design (RDD) was used to evaluate the changes in drug proportion and economic burden before and after the implementation of ZMDP, to provide policymakers with needle-targeted information.

### Data sources and sample selection

To assess the economic burden of stroke, we collected data on two aspects from 2016 to 2018. One was total data, including Health Financial Annual Report, Statistical Yearbook, China National Health Accounts Report, and Health Accounts Report, mainly from official sources. Another was patients’ medical expenses, which were collected from medical institutions by sampling survey.

The total data included the sum of income and basic expenditure subsidy from outpatient and inpatient services in different types of medical institutions in the region. The patients’ medical expenses come from the medical management system of each sample medical institution, including the detailed cost information and basic personal information of the patients in the medical institution, such as age, gender, and the fees of various medical services, etc.

The multistage stratification probability-proportional random sampling method was used in this study. Administrative regions and levels of economic development were stratified criteria. Different types of medical institutions were selected for each stratum, including general hospitals, specialized hospitals, traditional Chinese medicine hospitals, maternal and child health centers, and primary health institutions (including community health service centers, community health service stations, township health centers, etc). The sampling proportion was 1/3 to ensure that there was a representative sample [23]. Randomization was determined by a set of random sequences generated by a computer. (Appendix S1 in the **Online Supplementary Document**).

Stroke was identified based on the ICD-10 code, including I60-I64. I60-I62 represents ischemic stroke, I63 represents hemorrhagic stroke, and I64 doesn’t distinguish specific types. Samples with missing or abnormal data would be excluded, such as age, gender, and medical expenses. Finally, 223 187 stroke samples were collected, 71 453 in 2016, 78 679 in 2017, and 73 055 in 2018. Because patient identification data was anonymous, the local institutional review board waived the need for patient consent.

### Current curative expenditure (CCE) of stroke

CCE refers to the direct cost of treating a patient and does not include fixed capital formation costs. The CCE of stroke was calculated based on SHA 2011, including treatment income and treatment basic expenditure subsidy, divided into outpatient and inpatient parts. According to the ICD code, preventive services were excluded, and the proportion of stroke costs in the collected sample institutions was assessed. The basic treatment subsidy was determined by removing the preventive expenditure subsidy from the basic expenditure subsidy. The apportion coefficient was based on the sample institution. After several years of practice, SHA 2011 has become increasingly mature in China. The calculation method could be referred to in the previous study based on SHA 2011 [24].

### Quality control

The personnel involved in this study's data extraction and management had been engaged in this work for a long time and had received training from the National Health Commission of China many times. Data and results were reviewed by local health authorities and the National Health Commission of China.

### Regression discontinuity design (RDD)

For stroke, the outpatient cost was relatively single (almost all was drug cost). Therefore, we selected inpatient samples for analysis.

Since the sample institutions selected every year were not wholly consistent, we chose those institutions that participated in the survey during the three years. Thirteen medical institutions and 38 610 samples were included. The discharge date as the date point, July 1, 2017, was the implementation of the ZMDP. We eliminated those hospitalized before July 1, 2017, and discharged after July 1, 2017, because we couldn't be sure it had been affected by the policy. Finally, 38 373 samples were included in this study.

To accurately estimate the effect of ZMDP, this paper adopted sharp regression discontinuity. It is a quasi-experimental approach [25,26]. We converted the date into a specific value. Its absolute value represented the number of days away from the cutoff-point, where the left side was negative, and the right side was positive.

### Statistical analysis

Due to the non-normality of the expenditure distribution, we used median and interquartile to make descriptive statistics on outcome indicators for different years and subgroups. RDD analyzed the change of total expense per stay and drug proportion. Given their nonlinear distribution, we fitted a non-parametric local polynomial regression model using a fourth-order polynomial distribution of data before and after the cutoff-point [27]. The optimal bandwidth was automatically determined, where the BW type was mserd, and the Kernel was Triangular, giving more weight to observations closer to the threshold. The test results were revised, conventional variance estimators and robust variance estimators were performed to check the robustness. We varied the bandwidth to one-half and one-fourth of the data distribution across the cutoff-point in sensitivity analyses. Besides, subgroup analysis was also performed. All analyses were performed using STATA version 15.1 (StataCorp, College Station, TX, USA). We used the “rdplot” and “rdrobust” package to conduct the RDD. The level of significance was set at 0.05.

## RESULTS

### Stroke expenditure in 2016-2018

Based on SHA 2011, the CCE of stroke in Shanxi Province from 2016 to 2018 was 4374.69, 3727.22, and 3752.52 million CNY, respectively. Compared with 2016, the CCE decreased significantly in 2017 and rose slightly in 2018. The proportion of out-of-pocket (OOP) declined year by year, with a more significant decrease in 2017 compared to 2016, the same trend as CCE/GDP. About 90% of the cost of stroke occurred during hospitalization, and 60% in general hospitals. Although there was a slight increase in primary medical institutions, the overall expenditure was still less than 20%, and there was a decrease in 2018 compared with 2017. Different types of stroke costs were also different. Ischemic stroke accounts for most of the costs of stroke (**Table 1**).

**Table 1:** Distribution of stroke expenditure in 2016-2018 (million CNY)

Classification	2016	2017	2018
CCE	4374.69	3727.22	3752.52
CCE/GDP (%)	0.34	0.24	0.22
OOP (%)	36.71	28.36	23.53
Treatment (%):			
Outpatient	314.67 (7.19)	412.81 (11.08)	446.53 (11.9)
Inpatient	4060.02 (92.81)	3314.41 (88.92)	3305.99 (88.1)
Type of hospital (%):			
General	2714.74 (62.06)	2314.83(62.11)	2243.39 (59.78)
Traditional Chinese medicine	426.45 (9.75)	327.87 (8.80)	437.19 (11.65)
Special	731.70 (16.73)	429.90 (11.53)	517.27 (13.78)
Primary	501.80 (11.47)	654.62 (17.56)	554.67 (14.78)
Type of sStroke (%):			
Ischemic	3592.28 (82.12)	2910.46 (78.09)	2779.69 (74.08)
Hemorrhagic	767.52 (17.54)	666.58 (17.88)	869.28 (23.17)
Not specifically	14.89 (0.34)	150.18 (4.03)	103.56 (2.76)

CCE – current curative expenditure, GDP – gross domestic product, OOP – out-of-pocket

### Stroke expenditure in different age groups

**Figure 1:**
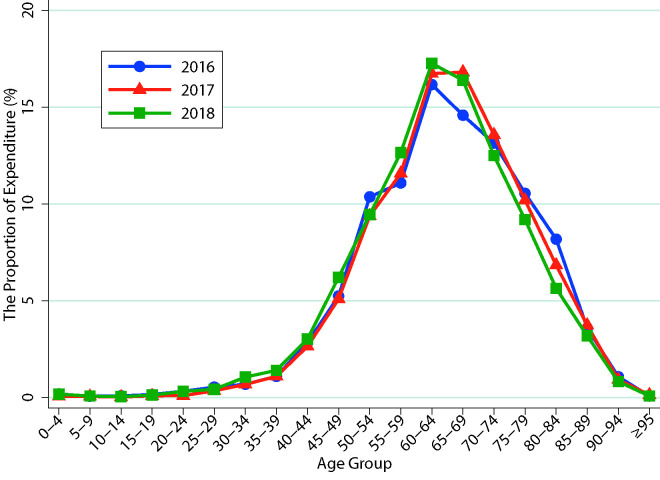
Age distribution of stroke expenditure in 2016-2018.

The proportion of stroke expenditure varied greatly among different age groups. In general, it began to rise significantly after the age of 40 and reached a peak between 60 and 70. Compared with 2016, there were differences in the 65-69 age group in 2017 and 2018. It was 14.59% in 2016, compared with 16.82% and 16.40% in 2017 and 2018. The 75-84 age group showed a year-on-year decline (**Figure 1**).

### Drug proportion and total expense of stroke expenditure

**Figure 2:**
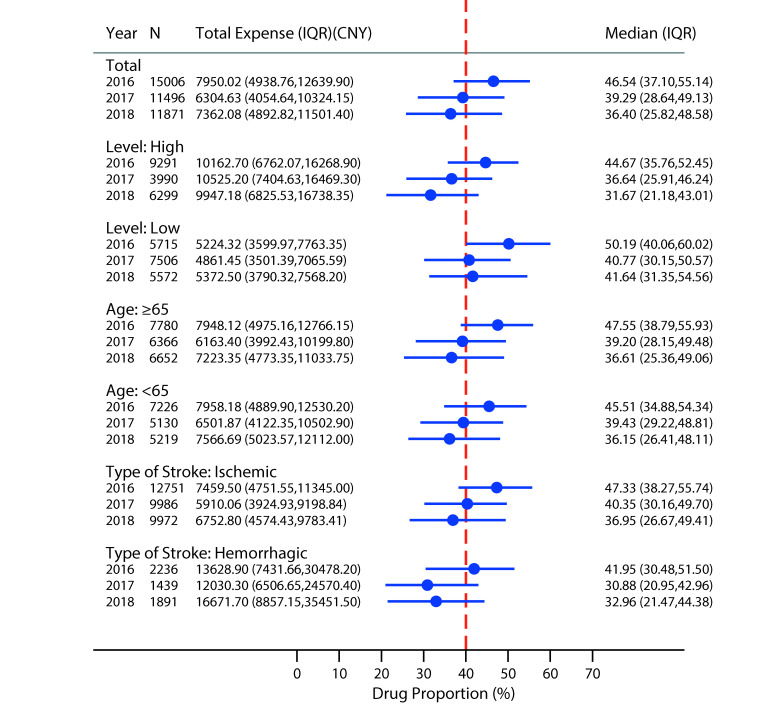
The change of total expense and drug proportion of stroke expenditure.

We selected 38 373 samples of hospitalization cost information, 15 006 in 2016, 11 496 in 2017, and 11 871 in 2018. The drug proportion decreased from 46.54% (interquartile range (IQR) = 37.10%, 55.14%) in 2016 to 36.40% (IQR = 25.82%, 48.58%) in 2018. High-level (Provincial and municipal level) medical institutions, the age group under 65 years old, and the ischemic stroke showed a decreasing trend year by year. The Low-level (District and county level) medical institutions and the hemorrhagic stroke group were inconsistent. From 2016 to 2017, the drug proportion all decreased significantly, both of which exceeded 10%. However, in 2018, the drug proportion did not decrease, increasing slightly, 1% in the medical institutions and 2% in the hemorrhagic stroke group (**Figure 2**).

The variation in total expense per stay was considerable. It was 7950.02 (IQR = 4938.76, 12 639.90) CNY in 2016, which decreased to 6304.63 (IQR = 4054.64, 40 324.15) CNY in 2017, and increased to 7362.08 (IQR = 4892.82, 11 501.40) CNY in 2018. From 2016 to 2018, all groups showed decreasing first and then increasing except for high-level medical institutions. In 2018, the expense of high-level medical institutions was 9947.18 (IQR = 6825.53, 16 738.35) CNY, lower than 2016. The total expense of stroke expenditure was significantly different in subgroups, higher in high-level medical institutions and hemorrhagic stroke. Hemorrhagic stroke in 2018 was the highest at 16 671.70 (IQR = 8857.15, 35 451.50) CNY (**Figure 2**).

### The effect of ZMDP

Density figures showed that the number of samples on both sides of the cutoff-point were symmetric, both for the whole and subgroups (Figure S1 and S2 in the **Online Supplementary Document**).

**Figure 3:**
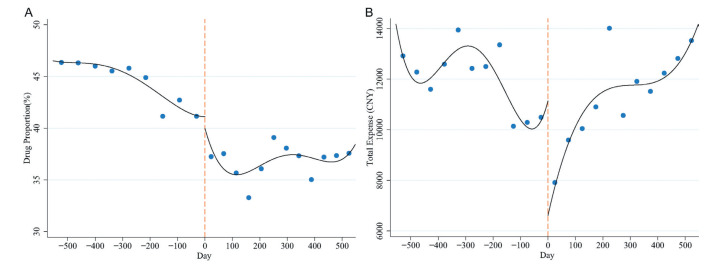
Zero-margin drug policy (ZMDP) for stroke. **Panel A.** Drug proportion. **Panel B.** Total expense.

For the drug proportion and total expense, the right side of the cutoff-point showed a significant decrease compared with the left side. However, different from the fact that the drug proportion remained stable after the decline (**Figure 3**, Panel A), the total expense showed an increase after the decrease (**Figure 3**, Panel B). No matter for drug proportion or total expense, although all subgroups showed down at the cutoff-point, the changing trend of each subgroup was different. The drug proportion in the low-level medical institutions continued to rise one year after implementing the policy, while its total expense fluctuated widely, declining rapidly at the end of 2018 (Figure S3 and S4 in the **Online Supplementary Document**).

With the implementation of the policy, the specific changes in drug proportion and total expense could be seen in **Table 2**. The drug proportion decreased by 2.76% (95% CI = 1.17%, 4.35%, *P* = 0.001), and the expense decreased by 4698.34 (95% CI = 3047.59, 6349.09, *P* < 0.001) CNY. The results of subgroup analysis showed that the drug proportion in high-level medical institutions, age ≥65, and ischemic stroke decreased more, and except for stroke type, the decrease rate was about twice that of the corresponding subgroup (4.50% vs 2.24%, 3.45% vs 1.86%). However, for total expense, the high-level medical institutions, age <65, and hemorrhagic stroke decreased more, with the high-level group 5.5 times more likely than the low-level group (9317.55 vs 1702.02 CNY), and hemorrhagic stroke six times more likely than ischemic stroke (14 772.44 vs 2463.04 CNY). Meantime, the drug proportion was not statistically significant in age <65 and hemorrhagic stroke (*P* > 0.05). Except for the drug proportion in the low-level group, the bias-corrected results and robustness tests were consistent with the conventional results. The results obtained by sensitivity analysis after changing the broadband were consistent with the robustness test results.

**Table 2:** Regression discontinuity estimates of the drug zero margin policy

Variables		Conventional			Bias-corrected			Robust		
**Coefficient**	**95% CI**	***P*-value**	**Coefficient**	**95% CI**	***P*-value**	**Coefficient**	**95% CI**	***P*-value**
**Drug proportion (%)**	**Level:**									
	High	-4.50	-7.34, -1.66	0.002	-4.53	-7.37, -1.69	0.002	-4.53	-7.96, -1.09	0.010
	Low	-2.24	-4.30, -0.19	0.033	-2.09	-4.15, -0.03	0.047	-2.09	-4.60, 0.43	0.104
	**Age:**									
	≥65	-3.45	-5.63, -1.26	0.002	-3.46	-5.65, -1.27	0.002	-3.46	-6.13, -0.79	0.011
	<65	-1.86	-4.06, 0.35	0.099	-1.77	-3.98, 0.43	0.115	-1.77	-4.46, 0.91	0.196
	**Type of stroke:**									
	Ischemic	-3.20	-4.89, -1.51	<0.001	-3.09	-4.78, -1.40	<0.001	-3.09	-5.15, -1.03	0.003
	Hemorrhagic	-2.79	-8.70, 3.11	0.354	-3.61	-9.51, 2.29	0.231	-3.61	-10.37, 3.15	0.295
	Total	-2.76	-4.35, -1.17	0.001	-2.75	-4.34, -1.16	0.001	-2.75	-4.69, -0.81	0.005
**Total expense (CNY)**	**Level:**									
	High	-9317.55	-12 733.49, -5901.60	<0.001	-9921.26	-13 337.20, -6505.31	<0.001	-9921.26	-13 812.43, -6030.08	<0.001
	Low	-1702.02	-2689.98, -714.05	0.001	-2015.53	-3003.50, -1027.56	<0.001	-2015.53	-3112.53, -918.52	<0.001
	**Age:**									
	≥65	-4586.51	-6513.76, -2659.26	<0.001	-5119.84	-7047.09, -3192.59	<0.001	-5119.84	-7258.92, -2980.76	<0.001
	<65	-5165.54	-7603.65, -2727.44	<0.001	-5592.68	-8030.79, -3154.58	<0.001	-5592.68	-8434.87, -2750.50	<0.001
	**Type of stroke:**									
	Ischemic	-2463.04	-3530.81, -1395.27	<0.001	-2756.03	-3823.80, -1688.26	<0.001	-2756.03	-3908.72, -1603.34	<0.001
	Hemorrhagic	-14 772.44	-22 264.72, -7280.17	<0.001	-15 472.21	-22 964.48, -7979.94	<0.001	-15 472.21	-24 312.40, -6632.02	0.001
	Total	-4698.34	-6349.09, -3047.59	<0.001	-5 109.58	-6760.33, -3458.83	<0.001	-5109.58	-6963.91, -3255.25	<0.001

CI – confidence interval

## DISCUSSION

The estimated risk of stroke was 39.3% in China, which is the highest in the world [2]. This study verified this conclusion from the perspective of the economic burden of disease. The results showed that in Shanxi Province of China, the annual direct medical expenses of stroke were about 4 billion CNY, the proportion of stroke in GDP decreased from 0.34% in 2016 to 0.22% in 2018. In particular, the implementation of ZMDP on drug prices has significantly reduced the total medical costs. The medical expenses of stroke mainly occurred in high-level general hospitals. The incidence of hemorrhagic stroke is low, but the cost of one time is much higher than that of ischemic stroke, and the total medical cost of ischemic stroke accounted for about 80% of all strokes.

Changes in the disease spectrum have shifted the global burden of disease from infectious to chronic diseases [1]. As a common chronic disease, stroke has a heavy economic burden in many countries and regions around the world [28]. Ageing populations exacerbate this phenomenon. The global lifetime risk of stroke increased by 8.9% from 1990 to 2016 [2]. In the United States, the average annual medical cost of stroke was US$40 billion [29], and in the United Kingdom was £8.9 billion [30]. Compared with the 4% of the total direct medical cost of stroke in developed countries [8], the results of this study showed that the economic burden of stroke in China was more serious (accounting for more than 5% of the total medical cost) [31], and general hospitals bear most of the medical expenses.

Previous studies have shown that inpatient services account for more than 90% of the health care costs of stroke patients [32,33], which is consistent with the findings of this study. Therefore, we have made further analysis of hospitalization expenses in Shanxi Province. Due to the difference in medical service costs, the average cost per stroke inpatient in China was lower than in developed countries, such as South Korea and Singapore [32,34]. For Shanxi Province, China, the hospitalization cost of stroke varies greatly among medical institutions of different levels. Average health costs per stay in lower-level institutions are half of those in higher-level institutions.

After implementing ZMDP, the proportion of OOP in stroke patients in Shanxi Province decreased from 36.71% to 23.53%. Both are lower than previously reported in northeast China [35]. We found that the ZMDP had different effects on different subgroups. As for the drug proportion, although some subgroups, such as low-level medical institutions and hemorrhagic stroke, showed a slight increase in 2018, the overall drug proportion showed a downward trend. However, for average health costs per stay, in the low-level medical institutions and hemorrhagic stroke groups, the costs were higher in 2018 than in 2016.

This study further verified the impact of ZMDP on the health costs of inpatients with stroke by RDD. After implementing the policy, all subgroups' costs showed a significant decrease, especially for high-level medical institutions and hemorrhagic stroke. At the same time, the proportion of drugs in high-level medical institutions also decreased most significantly among all subgroups. The results of this study on ZMDP were consistent with those reported in other provinces of China [36,37]. ZMDP decouples provider compensation from drug prescription and sales. It reduces drug costs and changes the structure of the hospital’s income to reflect the value of the health care labour market. However, hospitals will make up for the decrease in drug revenue by increasing services and products with a higher price and cost margin. Research shows that the cost of inpatient care doubled after implementing the ZMDP, which was driven by supply, instead of demand [15,38], which explains why expenditure rises significantly one year after implementing the policy. Hence, it is necessary to enforce policies to control the excessive growth of other costs while ensuring the quality of care.

Although the ZMDP seems to have achieved some success, the most effective to control the economic burden of stroke is prevention. Adopting a healthy diet, smoking cessation, and appropriate exercise can prevent more than 80% of strokes [39]. Although the elderly are the leading group of the current financial burden of stroke [40], the incidence and hospitalization trend of stroke is increasing in younger people [41]. This study showed little difference in hospitalization costs per stay among different age groups, reminding us of the importance of prevention for young people. The risk factors of stroke were not analyzed in this study, but they must be considered if you want to control better, such as multiple chronic diseases, obesity, environment, etc [28].

This study has some limitations. First, we were limited by the data available. As a result, it remains an open question to what extent stroke costs can be generalized to all of China. Second, our study only addressed the direct initial hospital costs of stroke, while indirect costs were not considered, underestimating the financial burden of stroke patients. Third, we only included patients with the first stroke diagnosis, excluding some patients with multiple diseases. Fourth, the implementation of policy is a long-term process. The time of this study is limited, so it can only accurately evaluate the effect of the policy at the beginning of its implementation.

## CONCLUSION

The economic burden of stroke patients in Shanxi Province, China, was severe. There were significant differences among different institutions, age groups, and stroke types. At the beginning of implementation, ZMDP had a noticeable effect on reducing drug proportion and hospitalization costs, but it needs to be observed for a longer time. According to regional and population characteristics, the Chinese government should continue to implement relevant policies to control the increase of stroke costs according to regional and population characteristics.

## Additional material


Online Supplementary Document
