# Supplementary material for: Will the zero-margin drug policy reduce the economic burden of stroke patients in China?
Source: J Glob Health. 2021 Sep 30;11:08007. doi: 10.7189/jogh.11.08007 (PMC8501452; doi:10.7189/jogh.11.08007)
Supplement: Online Supplementary Document [file jogh-11-08007-s001.pdf]

Online Supplementary Document

Whether the zero-margin drug policy reduce the economic burden of stroke patients in China?

Quan Fang, Degao Shang, Yunxia Zhang, Xinli Geng, Fang Liu, Qin Zhang, Xin Wang

Appendix S1. Sampling design

The sampling design was consistent for each year of 2016-2018. The field survey adopted multi-stage stratified cluster sampling. In the first stage, according to the sampling principles of economic and health development level, geographical location and information level in Shanxi Province, 4 sample cities were selected, namely Taiyuan, Changzhi, Yuncheng, and Xinzhou. In the second stage, four districts and counties in each city were selected as sample counties. A total of 16 districts and counties were selected. In the third stage, eight community health service centers or health centers were selected from each county. A total of 128 townships (including communities), 768 community service stations or village clinics were selected from six villages in each township or community; two outpatient departments and ten clinics were selected from each district and county. A total of 1088 monitoring institutions were selected. After the survey area was identified, samples were taken according to the level and classification of health institutions. Provincial public health institutions (disease control institutions,

maternal and child health institutions, health education institutions, etc.) were usually one and directly covered. General hospitals, traditional Chinese medicine hospitals according to the size of 1/2 of the institutions as samples; Specialized hospitals were selected according to the type, and one hospital was selected from each type on the premise of conforming to the sampling principle. Health institutions at prefectural and county levels were sampled according to the same principles.

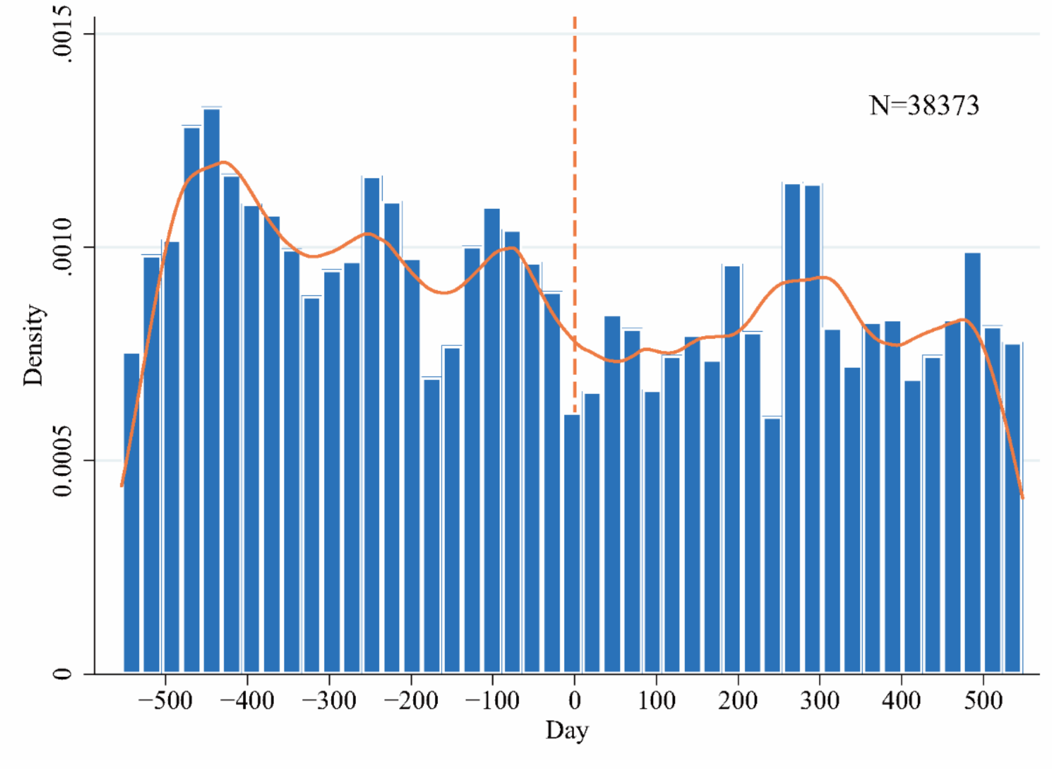

Figure S1. Density before and after implementation of ZMDP

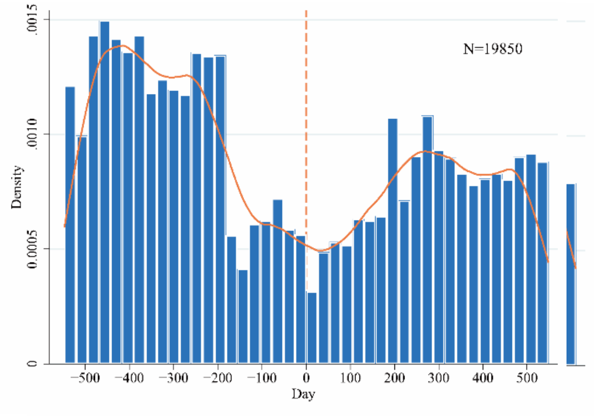
N=19850
Density
Day
.0015
.0010
0.0005
0
−500
−400
−300
−200
−100
0
100
200
300
400
500

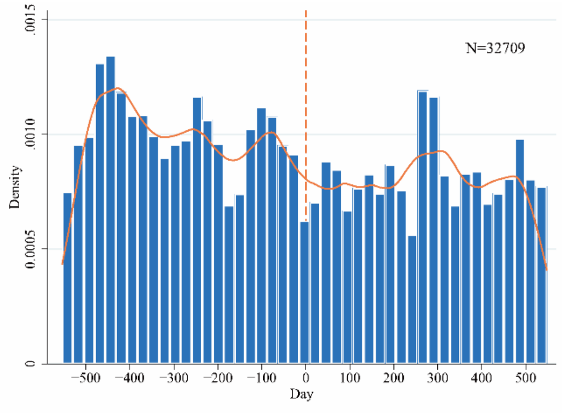
N=32709
Density
Day
.0015
.0010
0.0005
0
−500
−400
−300
−200
−100
0
100
200
300
400
500

(A)

(C)

(E)

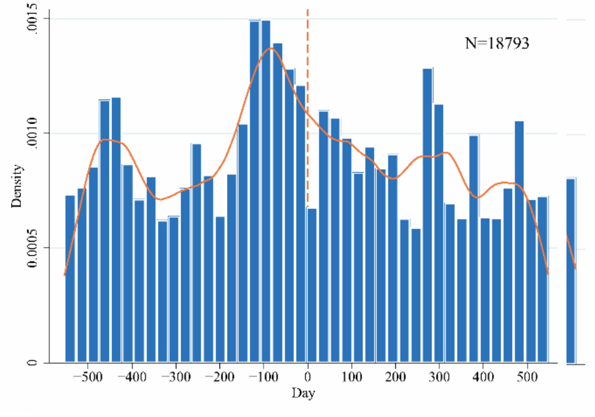

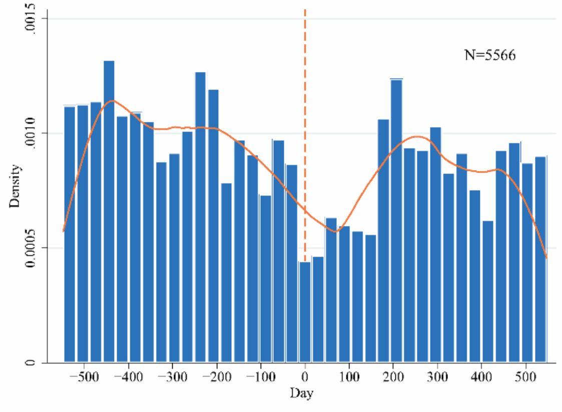

(B) (D) (F)

Figure S2. Density before and after implementation of ZMDP in different subgroups. (A) High-level institutions; (B) Low-level institutions; (C) Age≥65; (D) Age<65; (E) Ischemic stroke; (F) Hemorrhagic stroke.

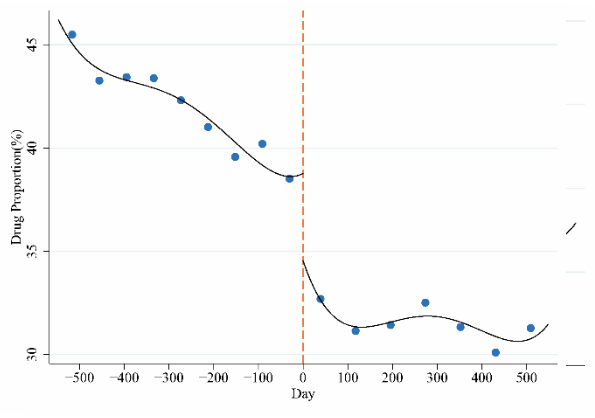
Drug Proportion(%)
45
40
35
30
−500 −400 −300 −200 −100 0 100 200 300 400 500
Day

(A)

(C)

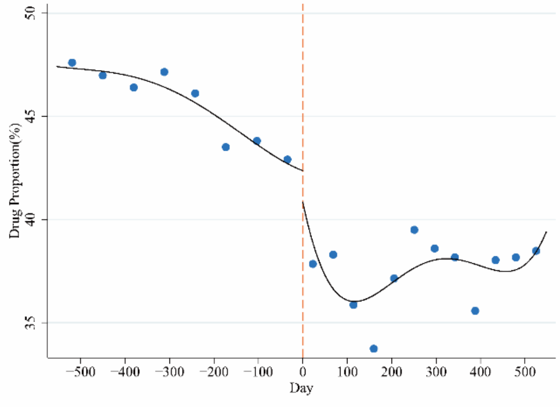
Drug Proportion(%)
50
45
40
35
−500 −400 −300 −200 −100 0 100 200 300 400 500
Day

(E)

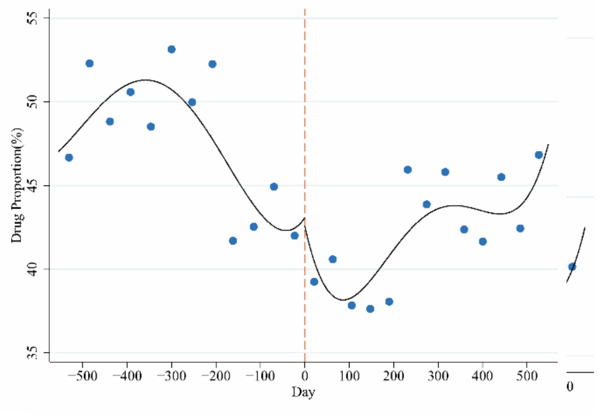

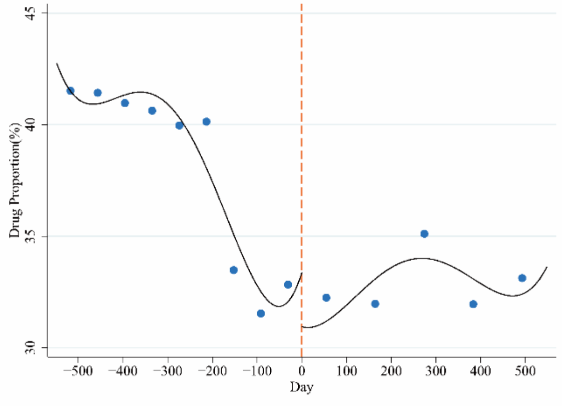

(B) (D) (F)

Figure S3. ZMDP for drug proportion in different subgroups. (A) High-level institutions; (B) Low-level institutions; (C) Age≥65; (D) Age<65; (E) Ischemic stroke; (F) Hemorrhagic stroke.

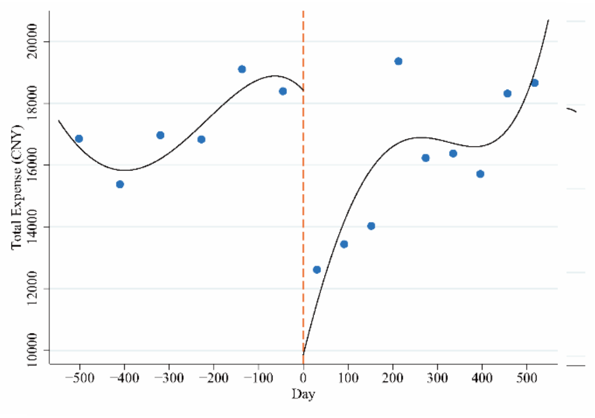

Total Expense (CNY)
10000
12000
14000
16000
18000
20000
−500
−400
−300
−200
−100
0
100
200
300
400
500
Day

(A)

(C)

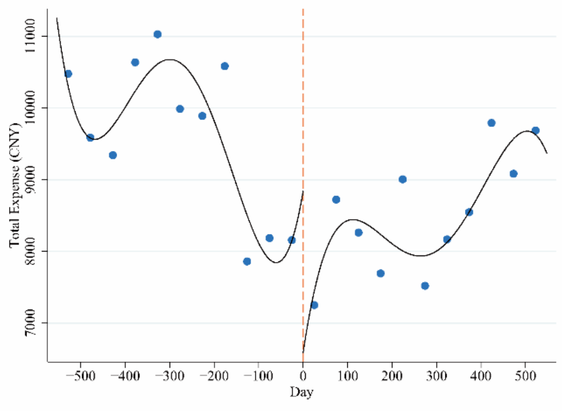

Total Expense (CNY)
7000
8000
9000
10000
11000
−500
−400
−300
−200
−100
0
100
200
300
400
500
Day

(E)

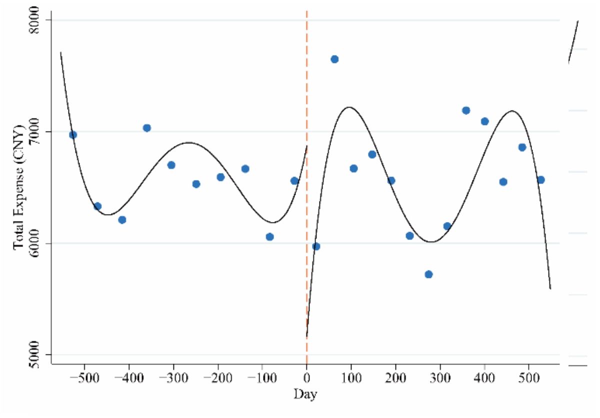

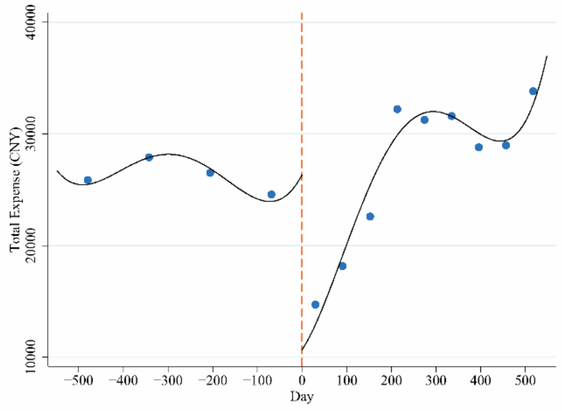

(B) (D) (F)

Figure S4. ZMDP for total expense in different subgroups. (A) High-level institutions; (B) Low-level institutions; (C) Age≥65; (D) Age<65; (E) Ischemic stroke; (F) Hemorrhagic stroke.
